# Causal associations of 25-hydroxyvitamin D with functional gastrointestinal disorders: a two-sample Mendelian randomization study

**DOI:** 10.1186/s12263-023-00734-1

**Published:** 2023-09-11

**Authors:** Senbao Xu, Qiuyan Luo, Jian He, Xiling Chen, Simin Li, Yang Bai

**Affiliations:** 1 Department of Gastroenterology, Nanfang Hospital, Southern Medical University, Guangzhou, 510515 China; 2 Department of Cytobiology, Southern Medical University, Guangzhou, 510515 China

**Keywords:** Genetic variants, Mendelian randomization, Vitamin D, 25-hydroxyvitamin D, Irritable bowel syndrome, Functional dyspepsia

## Abstract

**Background:**

Previous observational studies have shown associations between vitamin Ds and FGIDS[Including irritable bowel syndrome(IBS) and functional dyspepsia(FD)]. However, the association is controversial and the causality remains unknown. In this study, two-sample MR was cited to explore the causal effect on FGIDS caused by vitamin D level and serum 25-hydroxyvitamin D.

**Method:**

The GWASs of vitaminD and 25-hydroxyvitamin D, with 57–99 strongly related SNPs were all obtained from UK biobank. The GWASs of IBS and FD were obtained from FinnGen biobank with respectively 187,028 and 194,071 participants involved. Fixed-effect inverse variance weighted regression was used to evaluate causal estimates. Other statistical methods such as MR Egger, weighted median estimation, maximum likelihood estimation and penalty-weighted median estimation are also used to verify the accuracy of the main results.

**Results:**

Measuring by the IVW method, our research indicated that no causal relationship was detected between vitamin D intake and Functional gastrointestinal disorders [IVW, OR(vitamin D-IBS) = 0.909, 95% CI 0.789–1.053, *p* = 0.2017); OR(vitamin D-FD) = 1.0662, 95% CI 0.9182–1.2380, *p* = 0.4000]. As for serum 25-hydroxyvitamin D, no causal relationship was detected on FD(IVW, OR(25-hydroxyvitamin D-FD) = 0.9635, 95% CI 0.8039–1.1546, *p* = 0.6869). Nevertheless, a negative causal relationship was revealed between 25-hydroxyvitamin D and IBS(IVW, OR(25-hydroxyvitamin D-IBS) = 0.832, 95% CI 0.696–0.995, *p* = 0.0436). Sensitive analysis supported the main findings but did not suggest bias due to pleiotropy.

**Conclusions:**

Our Mendelian randomization analyses suggest a negative causal relationship between 25-hydroxyvitamin D and IBS. For each additional SD increase of genetically determined 25-hydroxyvitamin D levels, the risk of IBS decreased by 16.8%.

**Supplementary Information:**

The online version contains supplementary material available at 10.1186/s12263-023-00734-1.

## Introduction

Functional gastrointestinal disorders (FGIDs) are disorders of the digestive system where no biochemical abnormalities can be found to explain their symptoms, which often present with unexplained abdominal distension and abdominal pain related to defecation diarrhea [1]. FGIDs mainly include irritable bowel syndrome (IBS) and functional dyspepsia(FD) [2]. FGIDs are common diseases in modern society, a recent global survey of 54,127 adults in 26 countries initiated by the Rome Foundation reported that 43% of people met the symptoms of FGIDs [3]. Another retrospective study showed that almost all of the subjects who suffered from FGIDs had a comorbid disorder [4]. FGIDs are chronic and easy to relapse, which significantly reduce patients’ quality of life. Concurrently, repeating consultations, unnecessary medications as well as device examinations caused by FGIDs pose a significant burden on healthcare resources [5].

Vitamin D is an essential substance for health sustainability of human body [6], converted in the liver to 25-hydroxy vitamin D [25(OH)D], is the clinical biomarker of vitamin D status. Increasing evidences show that susceptibility to functional gastrointestinal disorders are influenced by vitamin D [7]. A retrospective study found that More than 50% of IBS subjects had vitamin D deficiency [8]. A study showed that patients with FD are characterized by an increased duodenal vitamin D receptor expression [9]. Another case–control study concluded that low levels of vitamin D remained a significant independent risk factor for the occurrence of intestinal motility disorder [10]. A recent study suggested that vitamin D supplementation improves only IBS-QoL scores but not IBS symptoms [11]. While several other studies suggested that vitamin D supplementation significantly improved symptoms of IBS [12–14]. These contradictory results may be caused by the unavoidably confounding factors in traditional clinical research. To explore the causality between vitamin D and FGIDs, Mendelian randomization(MR) is introduced in this research.

Mendelian randomization is a statistical method, which harnesses the properties of the genome to enable causal inference of biomarkers [15]. It aims to overcome the limitations of conventional medical research which can mislead investigator for reasons of confounding and reverse causation [16]. Traditional RCT analyses are not only expensive but also time-consuming. It is hard for those analyses to eliminate confounding factors leading to contradictory results. Mendelian randomization enables the use of a publicly available data from genome-wide association studies (GWAS) for both risk factor “exposures” and disease “outcomes” [16]. It relies on genetic variants qualifying the assumptions of an instrumental variable (IV), which means the IV will not directly impact the outcome but only serve as a bridge to the causal relationship between exposure and outcome. Since genetic variation remains stable throughout human lives, corresponding confounds are avoided in Mendelian randomization analysis.

In this study, two-sample MR was used to examine our hypothesis that the elevation of vitamin D level and serum 25-hydroxyvitamin D may be causally associated with risk of FGIDS.

## Methods

### Data source and open-GWAS statistics

The UK biobank (UKB) is a repository for biomedical data, containing information from approximately half a million participants [17]. The Integrative Epidemiology Unit (IEU) Open GWAS project, developed at the MRC Integrative Epidemiology Unit at the University of Bristol, sis mainly comprised of publicly available datasets [18]. This project now serves as an input source to conduct Mendelian randomization etc.

Four sets of data were chosen from the GWAS database in this study. Conditionally independent SNPs associated with 25-hydroxyvitamin D were acquired from UK Biobank, which contains a cohort of 443,734 individuals. In this GWAS, the average level of 25-hydroxyvitamin D, measured by the Diasorin assay, was determined to be 70 nmol/L (SD34.7 nmol/L) [19]. SNPs associated with Vitamin D were obtained from UK Biobank, encompassing 449,835 participants. In this GWAS, the mean vitamin D level was 48.5807 nmol/L (SD 21.1431 nmol/L). Two sets related to IBS and FD were both published in 2021, in which the former contains 4605 IBS European-descent patients, 182,423 European-descent controls, the letter includes 4376 and 189695 aforementioned patients and controls. The data above were all provided by the IEU Open GWAS database.

### SNPs selection and assumption

**Fig. 1:**
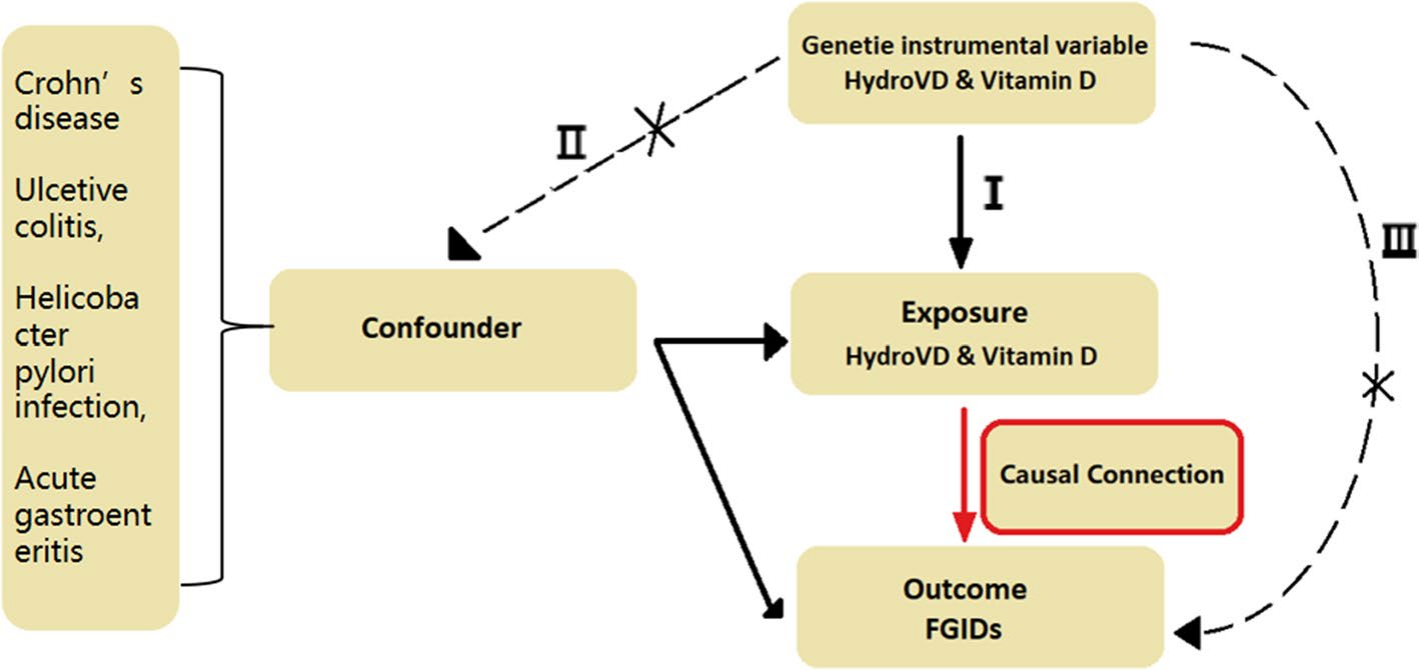
The flow diagram of the Mendelian randomization (MR) study. I The genetic instrumental variables (IVs) are strongly associated with 25-hydroxyvitamin D(HydroVD) and vitamin D. II The genetic IVs do not affect the outcome through the confounders. III The genetic IVs do not affect functional gastrointestinal disorders (FGIDs) directly, but only via indirect exposure

According to Fig. 1, MR analysis requires three core conditions: (I) the used variants are robustly associated with the exposure, which is called correlation hypothesis. (II) Variants we used are not associated with confounders of the risk factor–outcome association. (III) Variants does not directly affect the outcome, but affects via its effect on the risk factor of interest. Above the mentioned assumptions, a statistical significance level (*p* < 5 × 10 − 8) was strictly set to satisfy genome-wide strong associations. A specific threshold (*R*^2^ < 0.001)and mutation frequency (MAF ≥ 1%) were set for SNPs in order to attenuate linkage disequilibrium (LD). Finally, a total of 53–92 independent SNPs were identified as genetic instruments from the IEU Open GWAS database. These SNPs were selected by excluding other confounders such as Crohn’s disease, ulcerative colitis, *Helicobacter pylori* infection, acute gastroenteritis Through phenoscanner (http://www.phenoscanner.medschl.cam.ac.uk/). Additionally, IBS-related associated variants and FD-related associated variants were also excluded from the analysis.

### Statistical analysis

In this study, inverse variance weighted (IVW), MR-Egger, the maximum likelihood method and weighted median regression approaches were specifically chosen as analytical methods, providing more accurate evaluations by using the set of Wald ratio estimates, and testing and correcting as well as bias due to horizontal pleiotropic pathways. The web tool (https://shiny.cnsgenomics.com/mRnd) is used to calculate the statistical power.

The inverse-variance weighted (IVW) method, considered to be the most efficient analysis while encountering the absence of pleiotropic effects of IVs [20, 21], and thus adopted as the primary analysis method. However, since the estimate of IVW is a weighted average of the effects from each SNP, the result will be biased if any SNP shows horizontal pleiotropy. Therefore, only would the IVW model be applied when the heterogeneity was statistically significant (*p* < 0.05). Otherwise, the fixed effects model would be adopted.

The MR-Egger method and the penalized weighted median estimator provide statistical tests for presence of pleiotropic effects of the SNPs under analysis [22]. The Maximum likelihood method was used to gain the parameters of the associated probability density function for the database [23]. The causal relationship will be verifiable and convincing if the result of the leave-one-out analysis conformed to that of the IVW analysis. MR-PRESSO method was used to reduce horizontal pleiotropy by detecting and correcting outliers of SNPs in IVW analyses. The MR- PRESSO outlier test requires at least 50% of the genetic variants that are valid instrument and relied on InSIDE assumptions [24]. The method clusters the SNPs into groups according to the similarity of causal effects and returns the causal effect estimate based on the cluster with the largest number of SNPs. The number of distributions in MR-PRESSO analysis was set to 3000 while the significant threshold was set to 0.05.

The false discovery rate, based on the Benjamini–Hochberg method, was used to correct for multiple testing. The association with a nominal *P* value is < 0.05. But a Benjamini–Hochberg adjusted *P* value > 0.05 was considered suggestive while the association with a Benjamini–Hochberg adjusted *P* value < 0.05 was deemed significant.

All analyses were performed by R (version 4.1.1) and the Two Sample MR package.

## Results

### FinnGen biobank GWASs of vitamin D

The GWASs of vitamin D and 25-hydroxyvitamin D, with 57–99 strongly related SNPs were all obtained from UK biobank. The GWASs of IBS and FD were obtained from FinnGen biobank with respectively 187028 and 194071 participants involved (Table 1).

**Table 1:** The list of genome-wide summary association studies (GWAs) included in the Mendelian randomization (MR) study

Exposures	GWAS ID	Consortium	Sample size	No. of strongly related SNPs	Adjustment	Population
Vitamin D intake	ukb-d-30890_irnt	UK biobank	449,835	57	Crohn’s disease, Ulcerative colitis, *Helicobacter pylori* infection, Acute gastroenteritis	European
25-hydroxyvitamin D	ieu-b-4812	UK biobank	443,734	99		
Outcomes	GWAS ID	Consortium	Cases	Control	Population	
IBS	finn-b-K11_IBS	FinnGen biobank	4605	182,423	European	
FD	finn-b-K11_FUNCDYSP	FinnGen biobank	4376	189,695		

To testify the relationship between vitamin D-related genetic IVs with IBS and FD, reliable genetic IVs in vitamin D intake and serum vitamin D(25-hydroxyvitamin D) were identified in the FinnGen biobank database through open GWAS platforms (gwas.mrcieu.ac.uk). The identification procedure was executed by utilizing the functions of “extract_outcome_data” and “harmonise_data” and the 53–92 SNPs were extracted for the subsequent causality analysis (Tables 2 and 3).

**Table 2:** MR analysis results of exposure (including vitamin D intake and 25-hydroxyvitamin D) and irritable bowel syndrome (IBS)

Exposure	MR Method	No.SNP	β	SE	OR(95%CI)	*P*	Ajusted-*P*
Vitamin D intake	MR Egger	53	− 0.0664	0.1173	0.9357 (0.7434,1.1778)	0.57430	–
	Weighted median		− 0.1469	0.1082	0.86338 (0.70048,1.0641)	0.17458	–
	IVW(fixed effects)		− 0.09576	0.075005	0.90867 (0.76941,1.0731)	0.20168	0.4033
	Maximum likelihood		− 0.0959	0.0752	0.90859 (0.7840,1.0528)	0.2023	–
	Penalized-weighted median		− 0.14693	0.1084	0.86335 (0.6964,1.0703)	0.1741	–
	MR-PRESSO		− 0.09177	0.0845	0.9123 (0.8167,1.0192)	0.2825	–
25-hydroxyvitamin D	MR Egger	92	− 0.126	0.139	0.881 (0.671,1.158)	0.367	–
	Weighted median		− 0.189	0.140	0.828 (0.626,1.094)	0.175	–
	IVW (fixed effects)		− 0.184	0.0e1	0.832 (0.696,0.995)	0.0436	0.1744
	Maximum likelihood		− 0.185	0.091	0.832 (0.695,0.994)	0.0433	–
	Penalized-weighted median		− 0.189	0.135	0.828 (0.630,1.087)	0.160	–
	MR-PRESSO		− 0.17332	0.0934	0.8408 (0.7527,0.9393)	0.0667	–

*No. SNP* number of SNPs included in the analysis, *β* the regression coefficient based on vitamin D raising effect allele, *SE* standard error

*p* < 0.05 represents the causal link of vitamin D with IBS

**Table 3:** MR analysis results of exposure (including vitamin D intake and serum vitamin D) and functional dyspepsia (FD)

Exposure	MR Method	No.SNP	β	SE	OR(95%CI)	*P*	Ajusted-*P*
Vitamin D intake	MR Egger	53	− 0.051104	0.1045778	0.9501(0.7740,1.1663)	0.62716	−
	Weighted median		0.1229543	0.1184692	1.1308(0.8965,1.4264)	0.29933	−
	IVW (fixed effects)		0.0641415	0.076215	1.0662(0.9182,1.2380)	0.40001	−
	Maximum likelihood		0.0643745	0.0763783	1.0664(0.9182, 1.2387)	0.39931	0.53333
	Penalized-weighted median		0.136949	0.1203150	1.1467(0.9058,1.4517)	0.25501	−
	MR-PRESSO		0.0656	0.07594	1.0678(0.9558,1.1928)	0.39134	−
25-hydroxyvitaminD	MR Egger	92	− 0.17933	0.136803	0.8358( 0.6392,1.093)	0.1932	−
	Weighted median		− 0.087774	0.144605	0.915(0.6927, 1.211)	0.5439	−
	IVW (fixed effects)		− 0.03722	0.092349	0.9634(0.8039,1.155)	0.68693	0.6869
	Maximum likelihood		0.036860	0.092580	0.9638(0.8039, 1.156)	0.69052	−
	Penalized-weighted median		− 0.08717	0.14474	0.9165(0.6797,1.236)	0.54701	−
	MR-PRESSO		− 0.0325	0.0840	0.9680(0.8665,1.0813)	0.6996	−

*No. SNP* number of SNPs included in the analysis, *β* the regression coefficient based on vitamin D raising effect allele, *SE* standard error

*p* < 0.05 represents the causal link of fatty acid with FD

### IVW analysis

**Fig. 2:**
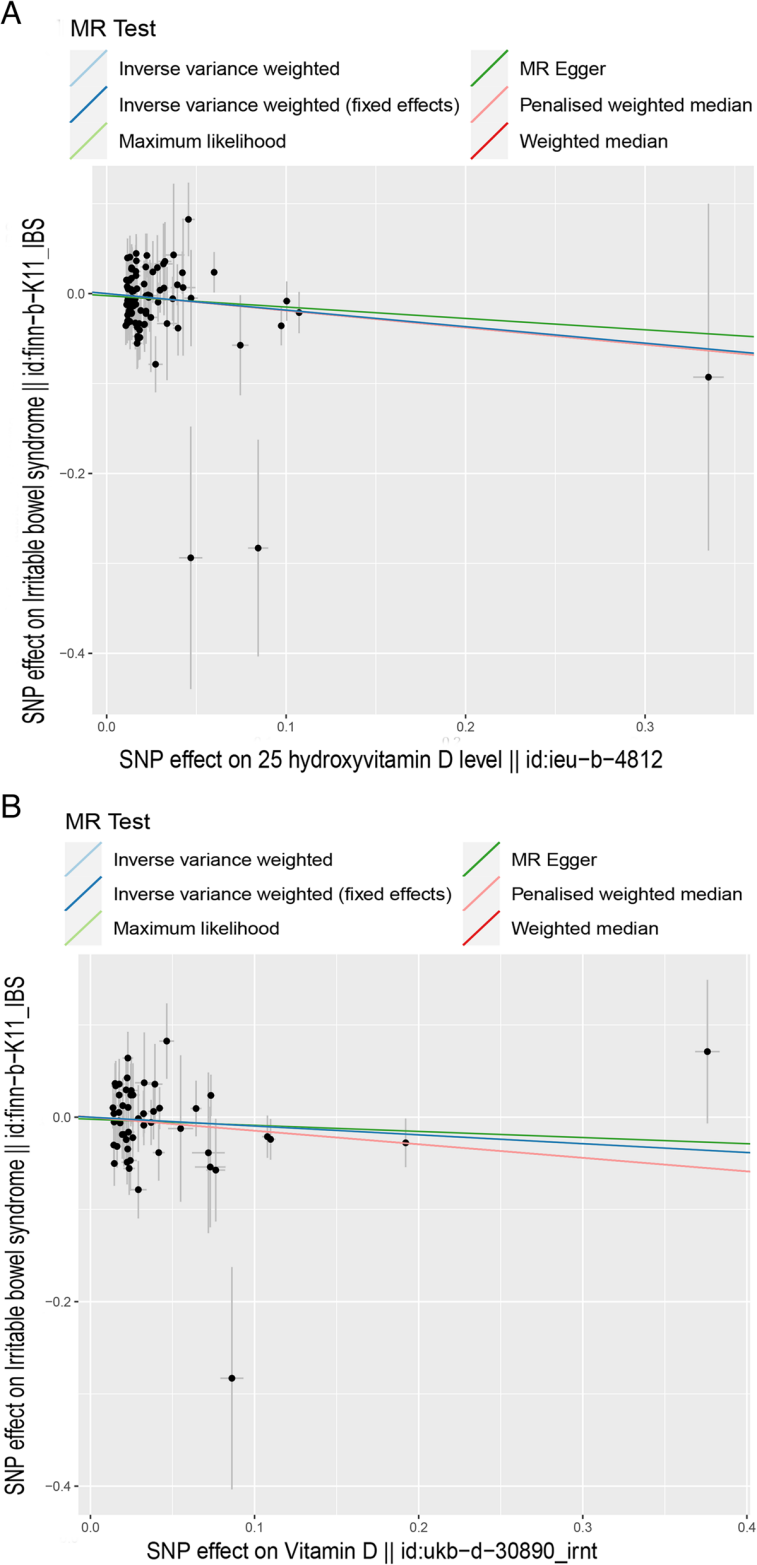
The scatter plot of vitamin (vitamin intake and 25-hydroxyvitamin D) and IBS. *X*-axis, the single nucleotide polymorphism (SNP) effect and standard errors (SEs) on each of the selected SNPs from vitamin genome-wide summary association study (GWAS) dataset. *Y*-axis, the SNP effect and SEs on IBS from IBS Genome-wide summary association study (GWAS) datasets. **A** Analysis of 25-hydroxyvitamin D and IBS. **B** Analysis of vitamin D intake and IBS

As illustrated in Tables 2 and 3, no causal relationship was detected between vitamin D intake and functional gastrointestinal disorders(including IBS and FD), with *p* values (≥ 0.05) measured by the IVW method. No causal relationship was detected between 25-hydroxyvitamin D and FD measured by the IVW method. Nevertheless, as shown in Table 2 and Fig. 2, a suggestive negative causal relationship was found between 25-hydroxyvitamin D and IBS[IVW, OR/95%CI: 0.832(0.696,0.995), *P* (0.0436), adjusted *P* value 0.1744; power = 0.80]. According to the IVW method, for each standard deviation (SD) increase in genetically determined 25-hydroxyvitamin D levels, the risk of IBS was found reduced by 16.8%.

### Supplementary and sensitivity analysis

In addition to the main IVW analysis methods, statistical methods such as MR Egger, weighted median estimation, maximum likelihood estimation, and penalty-weighted median estimation are also used to verify the accuracy of the main results. These complementary assays confirmed that vitamin D intake had no causal effect on IBS and FD or relationship between 25-hydroxyvitamin D and FD (*p* = 0.193, 0.544, 0.691, 0.547, respectively) (Tables 2 and 3). Negative causal relationship between 25-hydroxyvitamin D and IBS was detected by maximum likelihood method (*p* = 0.044). For each additional SD increase of genetically determined 25-hydroxyvitamin D levels, the risk of IBS decreased by 16.8%.

**Fig. 3:**
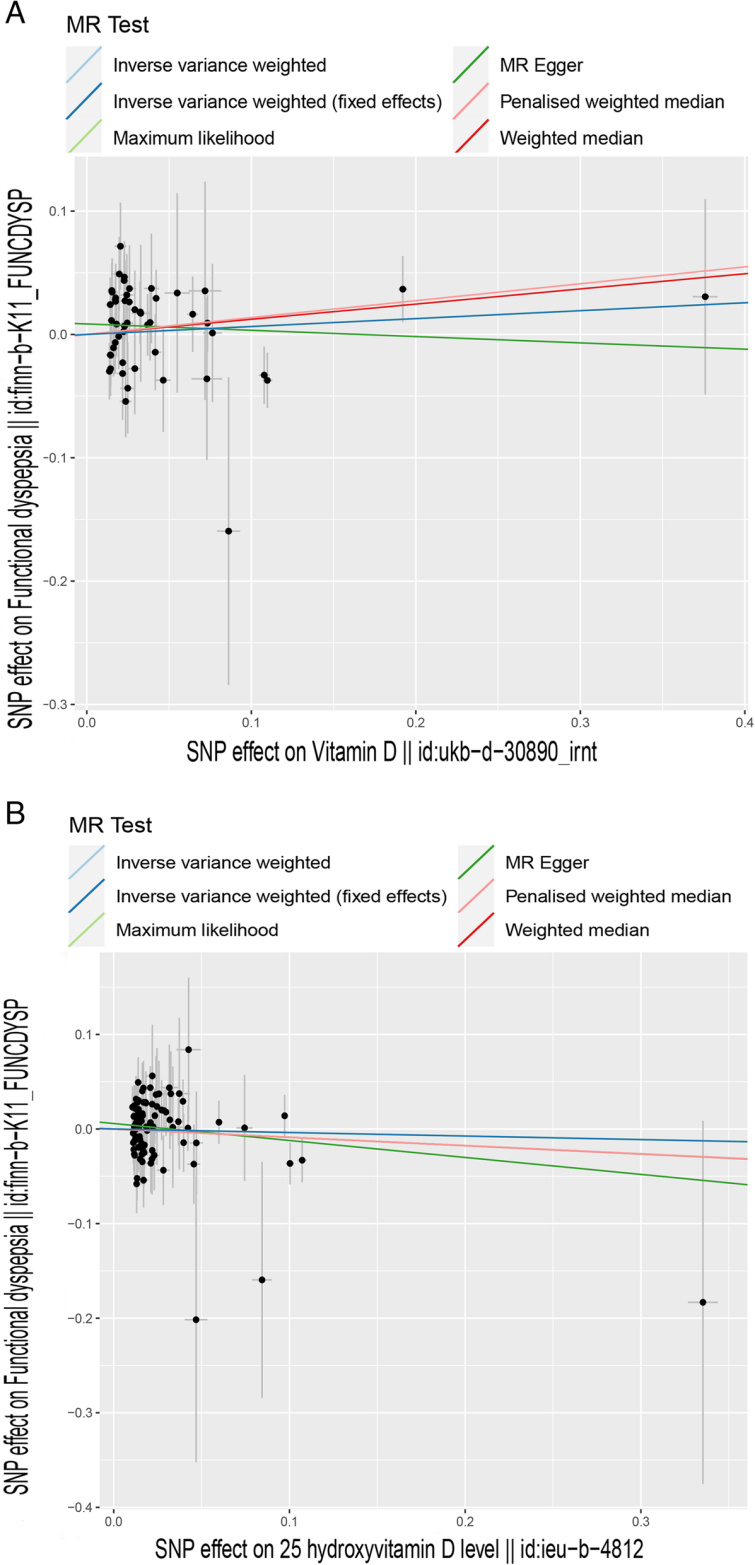
The scatter plot of vitamin Ds (vitamin D and 25-hydroxyvitamin D) and FD, *X*-axis, the single nucleotide polymorphism (SNP) effect and standard errors (SEs) on each of the selected SNPs from vitamin D genome-wide summary association study (GWAS) dataset. *Y*-axis, the SNP effect and SEs on FD from FD Genome-wide summary association study (GWAS) datasets. **A** Analysis of 25-hydroxyvitamin D and FD; **B** Analysis of vitamin D and FD

Scatter plots were used for visualizing the effect size of each MR Method (Figs. 2 and 3), forest maps for estimating individual SNPS for visualization results (Figures S1 and S2) and a funnel plot for showing the distribution balance of a single SNP effect (Figures S3 and S4). Concluding from these figures, the effects of each SNP and their distribution are in balance.

Heterogeneity was calculated by using Cochran’s* Q* statistic. As shown in Table 4, the effects of vitamin D intake (IVW, *p* = 0.084) and 25-hydroxyvitamin D (IVW, *p* = 0.328) on IBS revealed no statistically significant heterogeneity among the genetic instrumental variables. FD[Vitamin D(IVW, *p* = 0.448), 25-hydroxyvitamin D(IVW, *p* = 0.850)] produced the same statistical results. Therefore, the fixed effect IVW model was used for the primary MR Analysis.

**Table 4:** The heterogeneity test of vitamin D-related genetic variants in FGIDs Genome-wide summary association study (GWAS) datasets

Traits (outcome)	Exposure	Methods	*Q*	Q-dif	*P*
IBS	Vitamin D intake	MR Egger	66.41325911	51	0.072225879
		IVW	66.5877852	52	0.08393536
	25-hydroxyvitamin D	MR Egger	96.12027858	90	0.310
		IVW	96.45099224	91	0.328
FD	vitamin D	MR Egger	50.06724336	51	0.510672059
		IVW	52.65735198	52	0.448460363
	25-hydroxyvitamin D	MR Egger	75.11282569	90	0.870139247
		IVW	77.09561824	91	0.850433459

*p* < 0.05 is set as the significant threshold

**Fig. 4:**
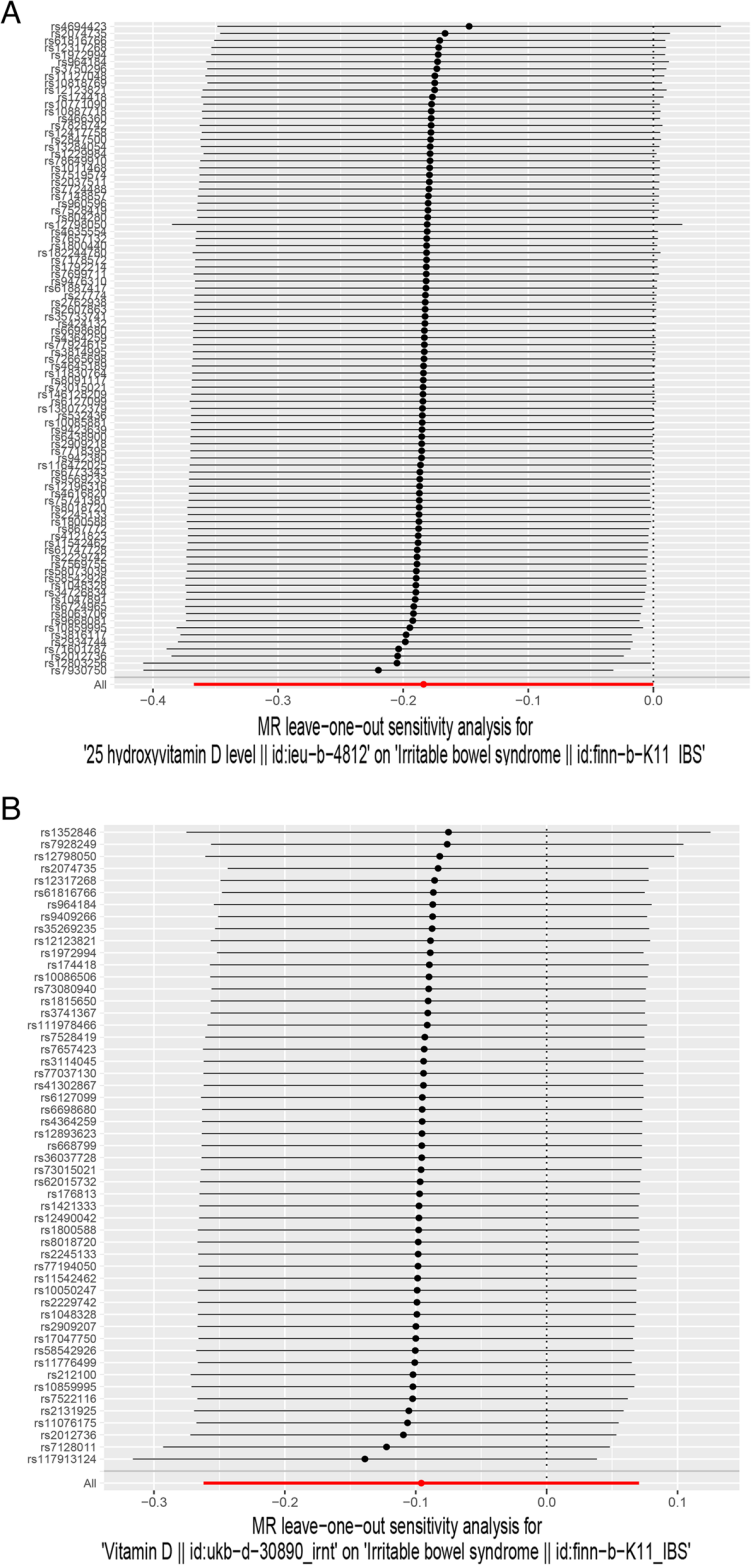
Leave-one-out analysis for the effect of vitamin Ds (25-hydroxyvitamin D and vitamin D)and IBS. **A** Analysis of 25-hydroxyvitamin D and IBS. **B** Analysis of vitamin D and IBS

**Fig. 5:**
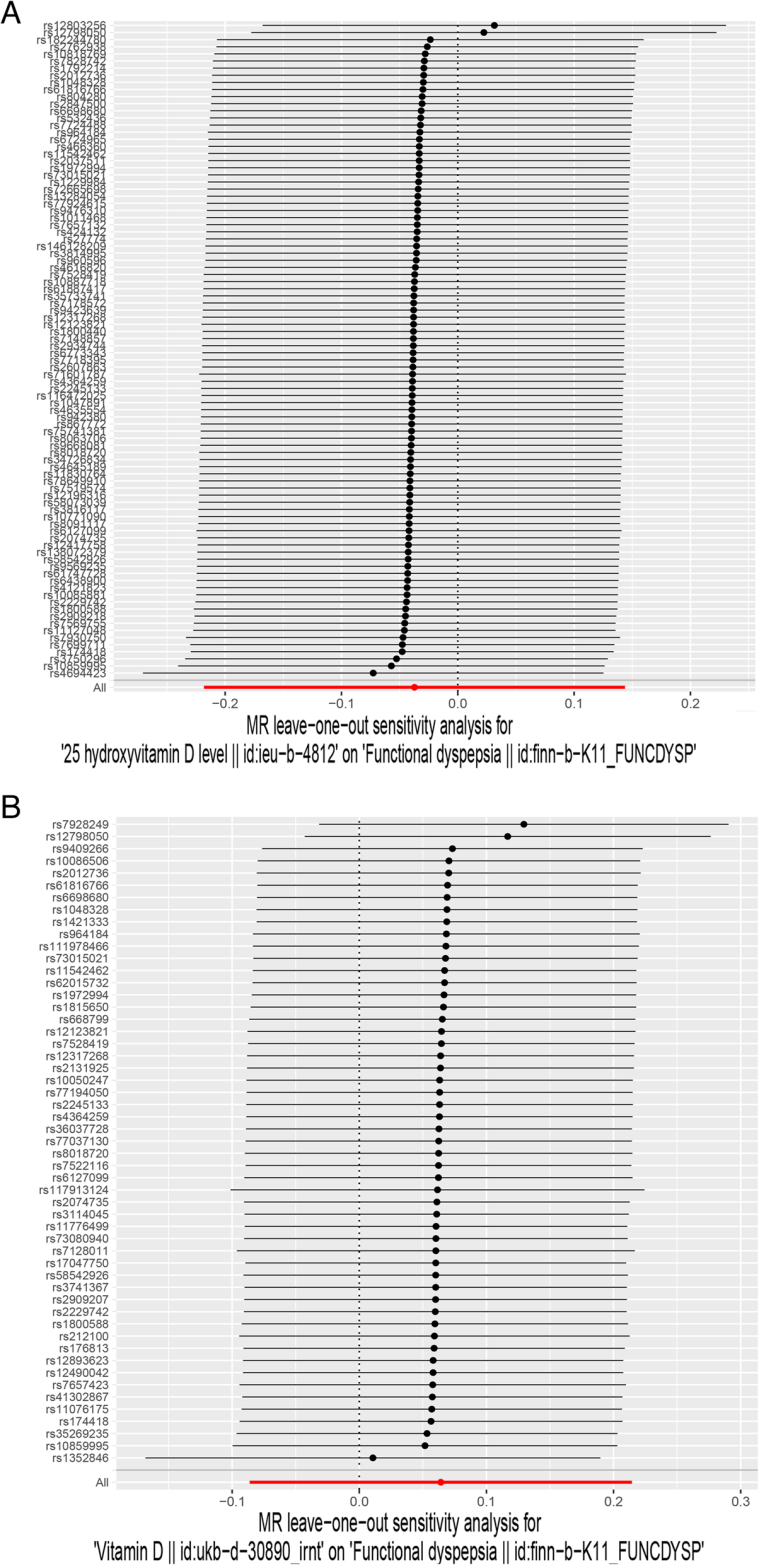
Leave-one-out analysis for the effect of vitamin Ds (25-hydroxyvitamin D and vitamin D)and IBS. **A** Analysis of 25-hydroxyvitamin D and FD. **B** Analysis of vitamin D and FD

The leave-one-out analysis was performed to assess the impact of individual SNPS on the final MR results. As shown in Figs. 4 and 5, the causal effects of the remaining vitamin D (vitamin D and 25-hydroxyvitamin D) on IBS and FD after successive omissions of the single SNPS were consistent with the results of the preliminary MR Study, indicating that no single SNPS played a significant role in the final results and the MR Study was stable and authentic.

Horizontal pleiotropy was tested to determine whether vitamin D-related genetic tool variants could lead to functional gastrointestinal disease(IBS and FD) through other potential pathways. The result of Table 5, indicating no horizontal pleiotropy presented in these MR Analyses illustrates the stability and reliability of the conclusions.

**Table 5:** The pleiotropic test of FAs genetic variants in inflammatory bowel disease (IBD) Genome-wide summary association study (GWAS) datasets

Traits (outcome)	Exposure	Egger_Intercept	se	*P*
IBS	Vitamin D	- 0.00216939	0.0059258	0.71581
	25-hydroxyvitamin D	- 0.00233	0.00420	0.57927
FD	Vitamin D	0.008493724	0.005277	0.1137062
	25-hydroxyvitamin D	0.00581	0.004126	0.162541

*se* standard error

*p* < 0.05 is set as the significant threshold

## Discussion

Functional gastrointestinal disorders, acting as a serious economic and social problem, are diseases with prevalence, uncertain cause and clinically challenges. Vitamin D plays an important role in the onset of FGIDs yet the effect is still unclear. 25-hydroxyvitaminD instead of vitamin D-intake was found out in this study with a negative causal effect on IBS. No causal relationship was detected between vitamin D (vitamin D intake and 25-hydroxyvitaminD) and FD.

After excluding potential confounders and other IBS- related characteristics from 99 strong 25-hydroxyvitamin D associated genetic IVs, comprising Crohn's disease, ulcerative colitis, Helicobacter Pylori infection and acute gastroenteritis, subsequently 94 SNPs were extracted from FinnGen biobank in the effect analysis of IBS.

Apart from the primary IVW analysis, maximum likelihood estimation, the other MR analysis method, also confirmed the increase of 25-hydroxyvitamin D caused by genetic factors can reduce the risk of IBS. No significant pleiotropic effect between 25-hydroxyvitamin D genetic variants and IBS was detected in the pleiotropic analysis (Table 2) and no statistically significant single SNP associated with the result was detected in the leave-one-out analysis (Fig. 3E). These results demonstrated that 25-hydroxyvitamin D genetic variants could affect the risk of IBS via 25-hydroxyvitamin D rather than other pathways.

Therefore, the existence of a causal relationship between 25-hydroxyvitamin D and IBS can be concluded from the study. No causal relationship was detected in the remaining groups in this study. The sensitive analysis, including the pleiotropic test, the leave-one-out analysis, and the heterogeneity examination cooperatively confirmed the robustness and reliability of the results. Supplementary MR methods also proved the validity of the study.

According to domestic and foreign studies, the effect of Vitamin D on FGIDs has been widely investigated. A retrospective study of pediatric patients with a confirmed diagnosis of IBS in the USA found that more than 50% of IBS subjects have vitamin D deficiency [8]. Another case–control study in Italy concluded that vitamin D low levels remain a significant independent risk factor for the occurrence of intestinal motility disorder [10]. A systematic review and meta-analysis considered all articles published before 4 April 2022, indicating no difference between vitamin D and placebo on the improvement of IBS.6 While other studies have reached very different conclusions. A random study shows that over the 6-month intervention period, a significantly greater alleviation to IBS symptoms was observed from the patients who keep receiving vitamin D, comparing with the placebo group [12]. Concurrently, other studies reported same results [13, 14]. The result of this MR study was consistent with that of the research conducted by Abbasnezhad A. By explorating the inhibiting occurrence of IBS. relationship between vitamin D and FGIDs, the rise in serum vitamin D levels were found.

In this MR study, the deficiencies of traditional observational studies were overcome and stronger conclusions were able to be drawn by utilizing a new analytic method.

Although the direct causes of FGIDs are currently unclear, several studies confirmed that immune system is associated [25]. A study using immunohistochemical method to analyze mast cells of IBS patients found that colonic mast cell infiltration and mediator release in proximity to mucosal innervation causing chronic inflammation and may contribute to abdominal pain perception in IBS patients [26]. Another research found that the number of mast cells are increasing in the colonic mucosa of children with IBS [27]. Given its potent regulator function in the immune system and anti-inflammatory effects [28], vitamin D can help improving low-grade mucosal inflammation and immune changes in patients afflicted with irritable bowel syndrome.

The key strengths of this study are as follows. Firstly, SNPs possessing robust association with exposure affecting the outcome indirectly were selected. Concurrently, the used SNPs are not associated with confounders of the risk factor–outcome association, which reinforced and upgraded the rationality and reliability of this study. Many SNPs were utilized as the IVs in this study, which usually substantially decreases the variance of the estimator. Secondly, the used GWAS data was obtained from patients of European descent, which avoids the bias of population stratification. Thirdly, altogether five MR Analysis methods were used to evaluate the consistency of the causal effect, which strengthened the credibility of our study.

Meanwhile, some weaknesses are inevitable in this study. Firstly, all participants are European, limiting the universality of the results. Secondly, this study provides only reliable evidence for the effect of vitamin D on the risk of having FGIDs while the effects of vitamin D on patients with FGIDs had not been explored. Thirdly, to provide sufficient evidence for this study, a series of experiments needed to be performed in the future to further explore the molecular mechanisms involved in vitamin D and FGIDs. Lastly, in the original literature on genetic variations, the authors did not provide cohort follow-up times. Therefore, the effect of follow-up time could not be judged though, it may still bias the results.

This MR study indicated that serum vitamin D is a significant protective factor of IBS instead of FD. However, this study is based on Vitamin D level determined by genetic variants, which could only be accounted for part of the IBS risk variation. Thus, a large-sample randomized controlled trail is still essential for clarifying the association between serum Vitamin D level and IBS.

## Conclusion

This MR study showed that 25-hydroxyvitamin D level had a negative causal effect on IBS instead of FD.

### Supplementary Information

**Additional file 1: Figure S1.** Forest plot and Funnel plot.

**Additional file 2: Figure S2.** Forest plot and Funnel plot.

**Additional file 3: Figure S3.** Forest plot and Funnel plot.

**Additional file 4: Figure S4.** Forest plot and Funnel plot.

**Additional file 5: Supplementary S5.** MR-PRESSO test of Exposures (vitamin D and 25-hydroxyvitamin D.

**Additional file 6.**

**Additional file 7.**

**Additional file 8.**

**Additional file 9.**

## Abbreviations

FGIDs: Functional gastrointestinal disorders
IBS: Irritable bowel syndrome
FD: Functional dyspepsia
MR: Mendelian randomization
IVW: Inverse variance weighted
UKB: The UK biobank
GWAS: Genome-wide association studies
IV: Instrumental variable
RCT: Randomized controlled trial
IEU: Integrative Epidemiology Unit
LD: Linkage disequilibrium
SNP: The single nucleotide polymorphism

## References

[1] Drossman DA (2016). Functional gastrointestinal disorders: history, pathophysiology, clinical features and Rome IV. Gastroenterology.

[2] Schmulson MJ, Drossman DA (2017). What is new in Rome IV. J Neurogastroenterol Motil.

[3] Sperber AD, Bangdiwala SI, Drossman DA, Ghoshal UC, Simren M (2021). Worldwide prevalence and burden of functional gastrointestinal disorders, results of Rome Foundation Global Study. Gastroenterology.

[4] Chelimsky G, Safder S, Chelimsky T (2012). FGIDs in children are associated with many nonpsychiatric comorbidities: the tip of an iceberg?. J Pediatr Gastroenterol Nutr.

[5] Black CJ, Drossman DA, Talley NJ, Ruddy J (2020). Functional gastrointestinal disorders: advances in understanding and management. Lancet.

[6] Zmijewski MA (2019). Vitamin D and human health. Int J Mol Sci.

[7] Huang H, Wang C, Lin W, Zeng Y, Wu B (2022). A population-based study on prevalence and predisposing risk factors of infant functional gastrointestinal disorders in a single center in Southern Fujian. Front Pediatr.

[8] Nwosu BU, Maranda L, Candela N (2017). Vitamin D status in pediatric irritable bowel syndrome. PLoS One.

[9] Beeckmans D, Riethorst D, Augustijns P, Vanuytsel T, Farré R, Tack J (2018). Altered duodenal bile salt concentration and receptor expression in functional dyspepsia. United European Gastroenterol J.

[10] Panarese A, Pesce F, Porcelli P, Riezzo G, Iacovazzi PA, Leone CM (2019). Chronic functional constipation is strongly linked to vitamin D deficiency. World J Gastroenterol.

[11] Abuelazm M, Muhammad S, Gamal M, Labieb F, Amin MA, Abdelazeem B (2022). The effect of vitamin D supplementation on the severity of symptoms and the quality of life in irritable bowel syndrome patients: a systematic review and meta-analysis of randomized controlled trials. Rev Nutr.

[12] Abbasnezhad A, Amani R, Hajiani E, Alavinejad P, Cheraghian B, Ghadiri A (2016). Effect of vitamin D on gastrointestinal symptoms and health-related quality of life in irritable bowel syndrome patients: a randomized double-blind clinical trial. Neurogastroenterol Motil.

[13] El Amrousy D, Hassan S, El Ashry H, Yousef M, Hodeib H (2018). Vitamin D supplementation in adolescents with irritable bowel syndrome: is it useful? A randomized controlled trial. Saudi J Gastroenterol.

[14] Sikaroudi KM, Mokhtare M, Janani L, Faghihi Kashani AH, Masoodi M, Agah S (2020). Vitamin D3 supplementation in diarrhea-predominant irritable bowel syndrome patients: the effects on symptoms improvement, serum corticotropin-releasing hormone, and interleukin-6—a randomized clinical trial. Complement Med Res.

[15] Bowden J, Holmes MV (2019). Meta-analysis and Mendelian randomization: a review. Res Synth Methods.

[16] Davey Smith G, Hemani G (2014). Mendelian randomization: genetic anchors for causal inference in epidemiological studies. Hum Mol Genet.

[17] Sudlow C, Gallacher J, Allen N, Beral V, Burton P, Danesh J, Downey P (2015). UK biobank: an open access resource for identifying the causes of a wide range of complex diseases of middle and old age. PLoS Med.

[18] Hemani G, Zheng J, Elsworth B, Wade KH, Haberland V, Baird D, Laurin C (2018). The MR-Base platform supports systematic causal inference across the human phenome. Elife.

[19] De La Barrera B, Manousaki D (2023). Serum 25-Hydroxyvitamin D levels and youth-onset type 2 diabetes: a two-sample Mendelian randomization study. Nutrients.

[20] Burgess S, Small DS, Thompson SG (2017). A review of instrumental variable estimators for Mendelian randomization. Stat Methods Med Res.

[21] Bowden J, Davey Smith G, Haycock PC, Burgess S (2016). Consistent estimation in Mendelian randomization with some invalid instruments using a weighted median estimator. Genet Epidemiol.

[22] Burgess S, Thompson SG (2017). Interpreting findings from Mendelian randomization using the MR-Egger method. Eur J Epidemiol.

[23] Milligan BG (2003). Maximum-likelihood estimation of relatedness. Genetics.

[24] Verbanck M, Chen CY, Neale B (2018). Detection of widespread horizontal pleiotropy in causal relationships inferred from Mendelian randomization between complex traits and diseases. Nat Genet.

[25] Barbara G, Cremon C, Carini G, Bellacosa L, Zecchi L, De Giorgio R (2011). The immune system in irritable bowel syndrome. J Neurogastroenterol Motil.

[26] Barbara G, Stanghellini V, De Giorgio R, Cremon C, Cottrell GS, Santini D, Pasquinelli G (2004). Activated mast cells in proximity to colonic nerves correlate with abdominal pain in irritable bowel syndrome. Gastroenterology.

[27] Di Nardo G, Barbara G, Cucchiara S, Cremon C, Shulman RJ, Isoldi S, Zecchi L (2014). Neuroimmune interactions at different intestinal sites are related to abdominal pain symptoms in children with IBS. Neurogastroenterol Motil.

[28] Mahon BD, Wittke A, Weaver V, Cantorna MT (2003). The targets of vitamin D depend on the differentiation and activation status of CD4 positive T cells. J Cell Biochem.

