# Supplementary figures and images for: Causal associations of 25-hydroxyvitamin D with functional gastrointestinal disorders: a two-sample Mendelian randomization study
Source: Genes Nutr. 2023 Sep 11;18:14. doi: 10.1186/s12263-023-00734-1 (PMC10494327; doi:10.1186/s12263-023-00734-1)

# MR Method

- Inverse variance weighted
- MR Egger

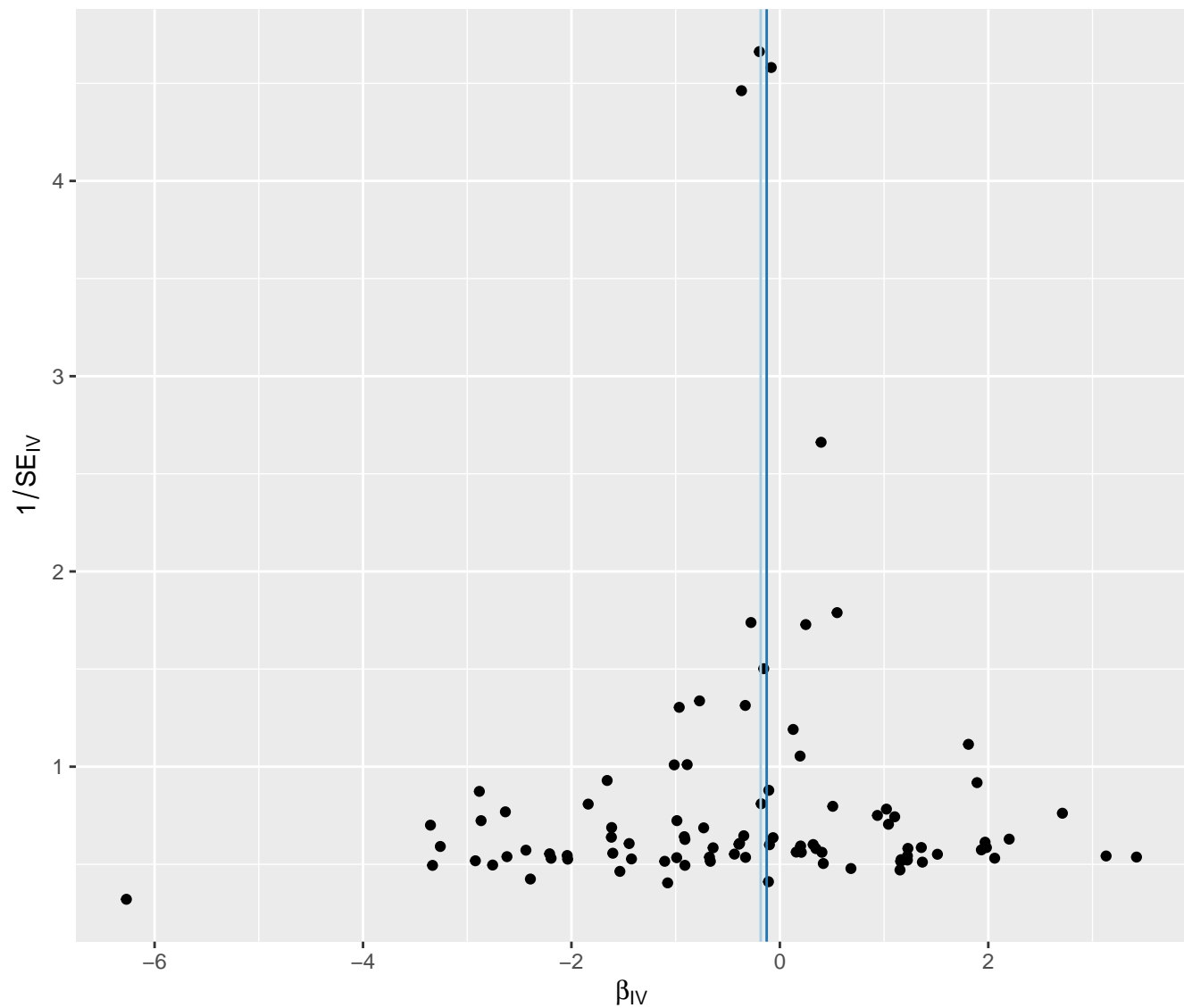

Supplement: Supplementary file 1 — Additional file 1: Figure S1. Forest plot and Funnel plot. [file 12263_2023_734_MOESM1_ESM.zip › Figure S1(Funnel plot) .pdf]

All – MR Egger  
All – Inverse variance weighted

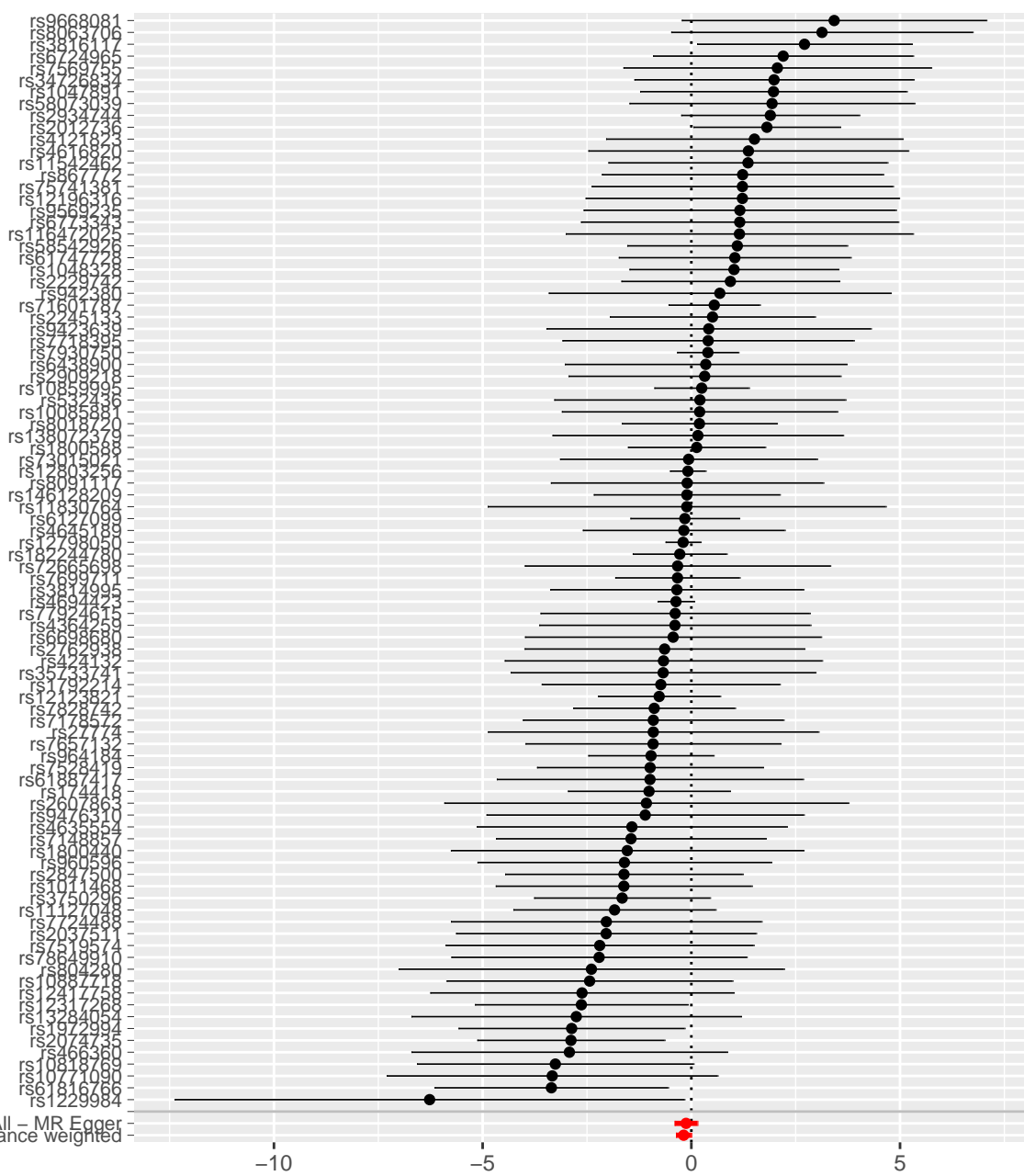

Supplement: Supplementary file 2 — Additional file 2: Figure S2. Forest plot and Funnel plot. [file 12263_2023_734_MOESM2_ESM.zip › Figure S2(Forest plot).pdf]

# MR Method

- Inverse variance weighted
- MR Egger

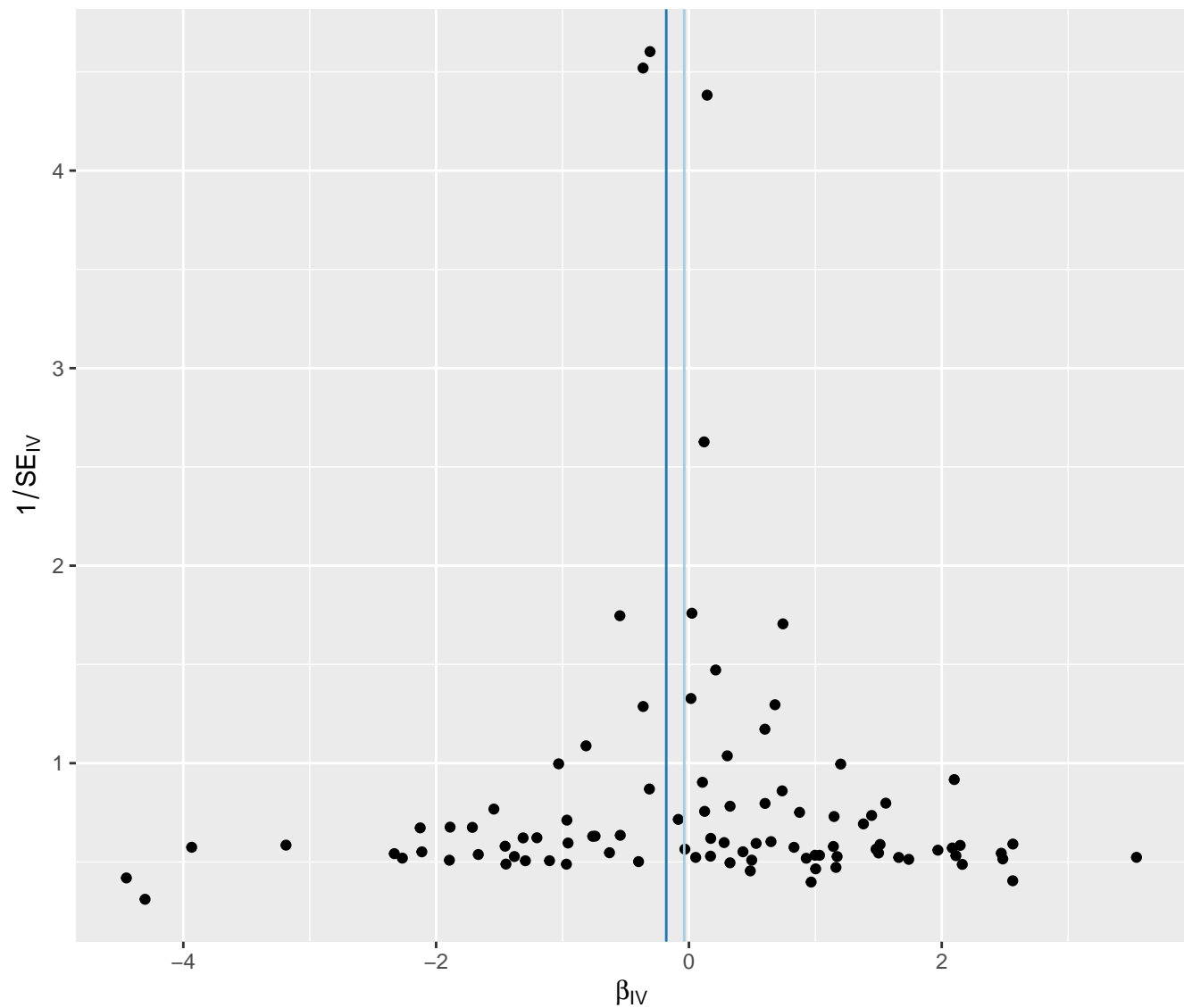

Supplement: Supplementary file 2 — Additional file 2: Figure S2. Forest plot and Funnel plot. [file 12263_2023_734_MOESM2_ESM.zip › Figure S2(Funnel plot) .pdf]

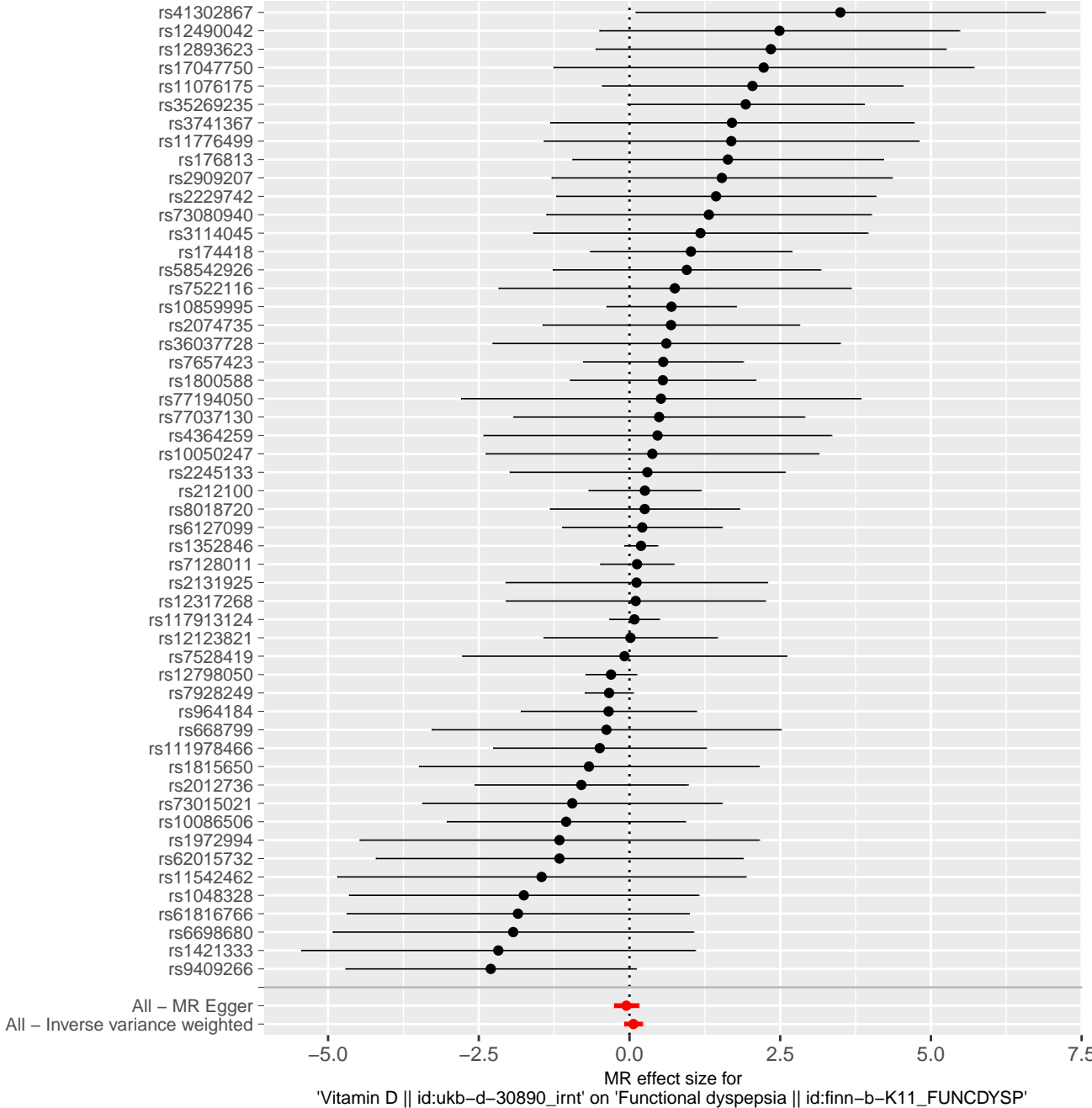

Supplement: Supplementary file 3 — Additional file 3: Figure S3. Forest plot and Funnel plot. [file 12263_2023_734_MOESM3_ESM.zip › Figure S3(Forest plot).pdf]

# MR Method

- Inverse variance weighted
- MR Egger

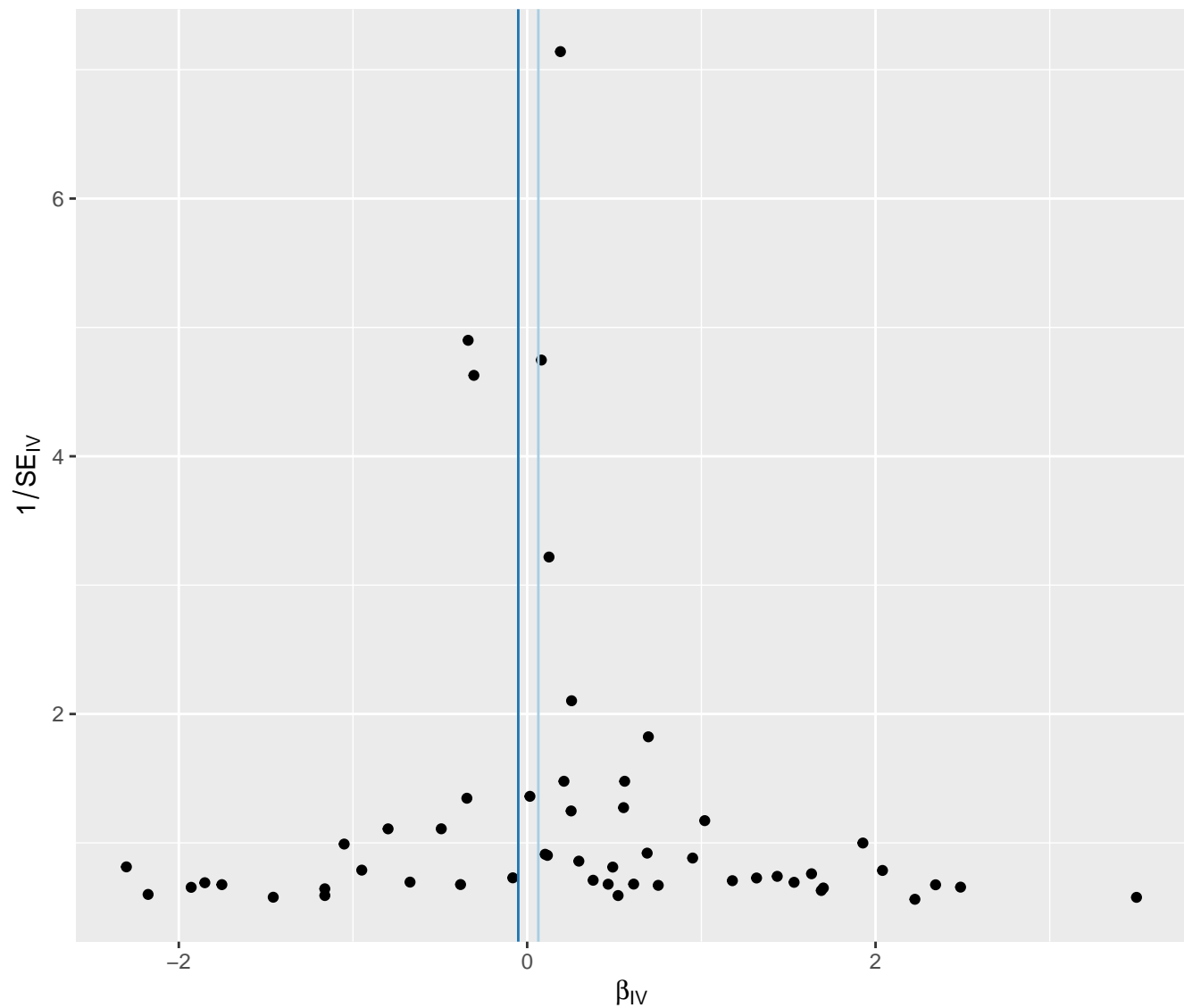

Supplement: Supplementary file 3 — Additional file 3: Figure S3. Forest plot and Funnel plot. [file 12263_2023_734_MOESM3_ESM.zip › Figure S3(Funnel plot).pdf]

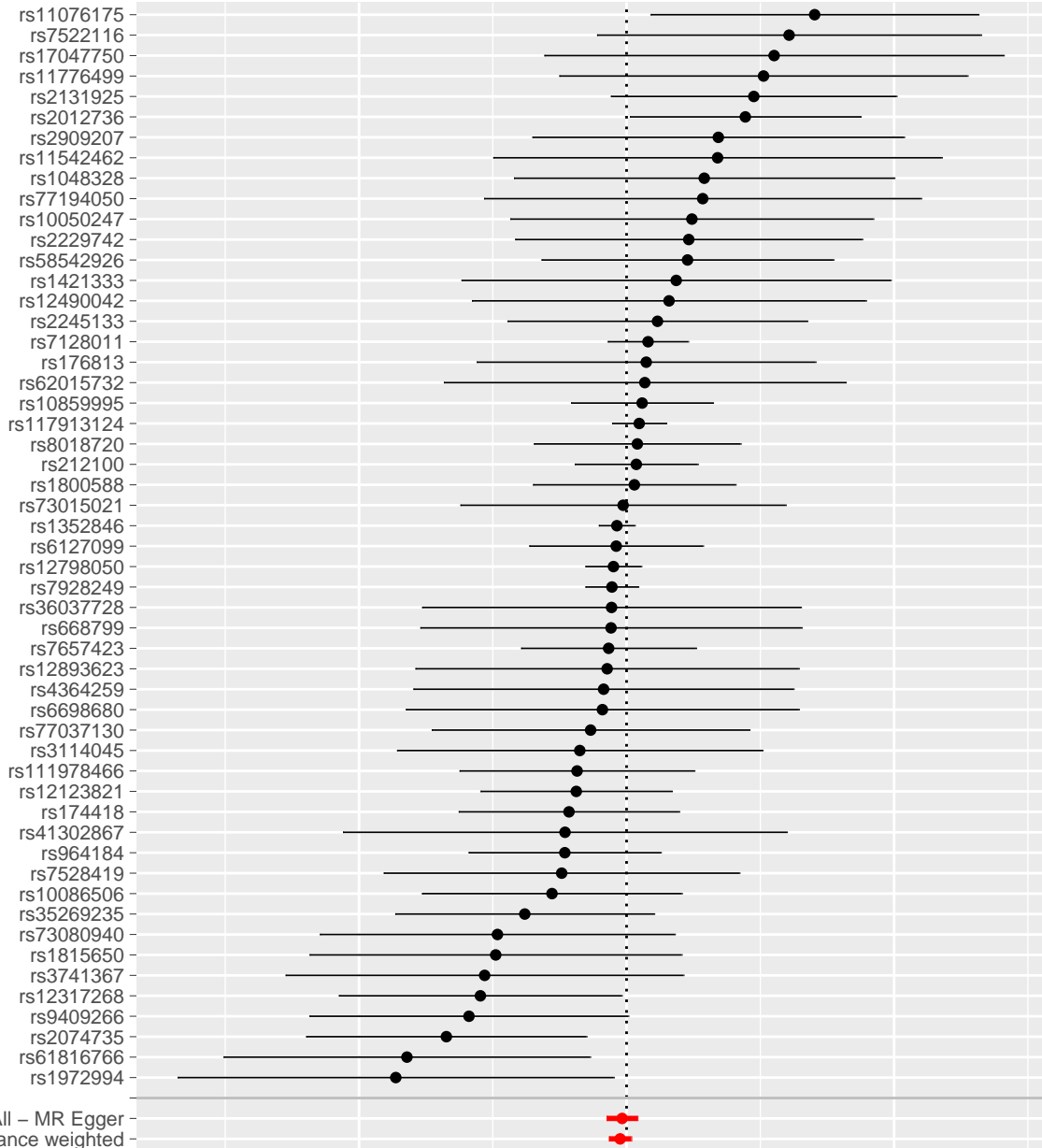

MR effect size for  
'Vitamin D || id:ukb-d-30890\_irnt' on 'Irritable bowel syndrome || id:finn-b-K11\_IBS'

Supplement: Supplementary file 4 — Additional file 4: Figure S4. Forest plot and Funnel plot. [file 12263_2023_734_MOESM4_ESM.zip › Figure S4(Forest plot).pdf]

# MR Method

- Inverse variance weighted
- MR Egger

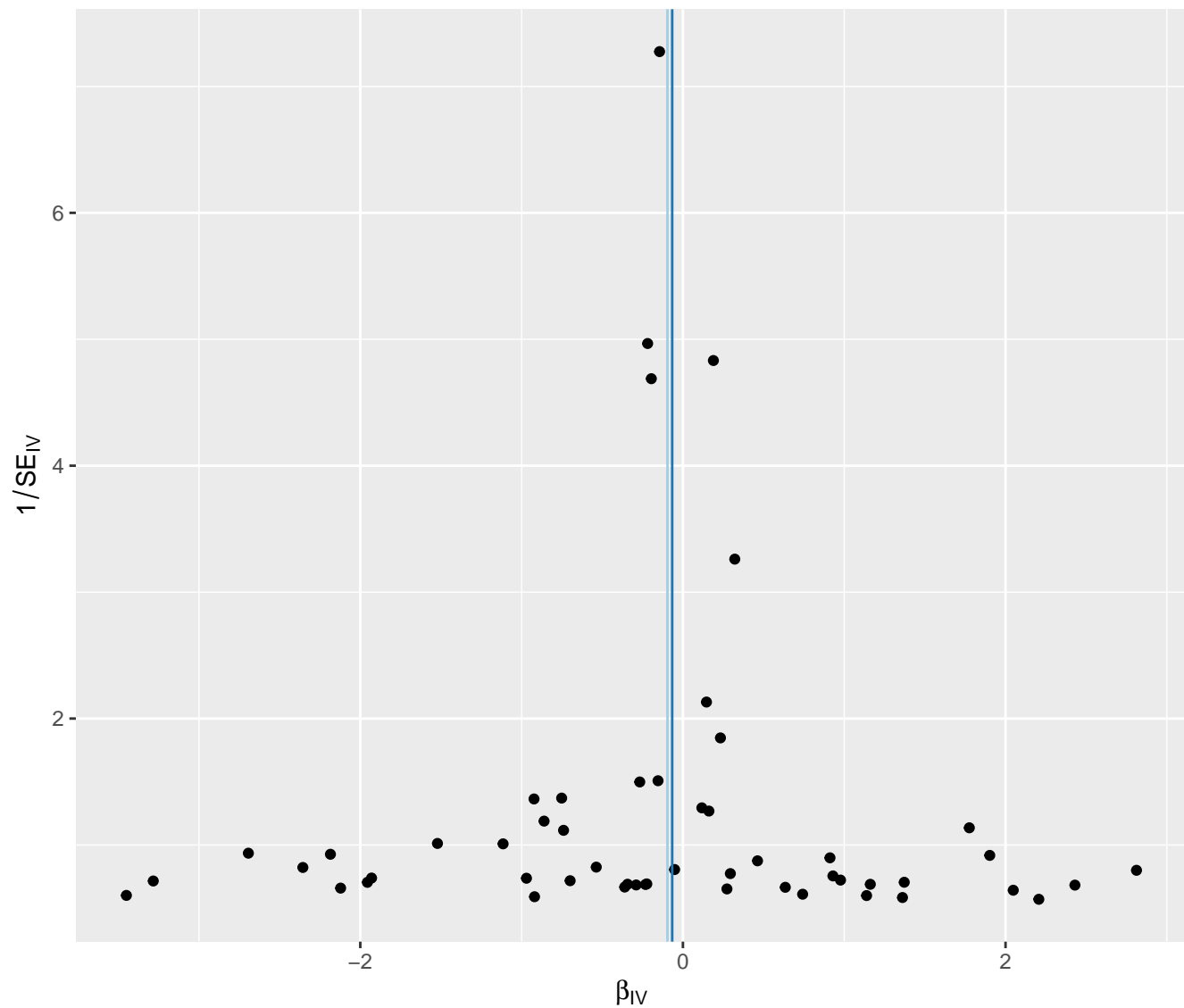

Supplement: Supplementary file 4 — Additional file 4: Figure S4. Forest plot and Funnel plot. [file 12263_2023_734_MOESM4_ESM.zip › Figure S4(Funnel plot).pdf]
