# Supplementary material for: Causal associations of 25-hydroxyvitamin D with functional gastrointestinal disorders: a two-sample Mendelian randomization study
Source: Genes Nutr. 2023 Sep 11;18:14. doi: 10.1186/s12263-023-00734-1 (PMC10494327; doi:10.1186/s12263-023-00734-1)
Supplement: Supplementary file 1 — Additional file 1: Figure S1. Forest plot and Funnel plot. [file Figure S1(Forest plot).pdf › Figure S1(Forest plot).pdf]

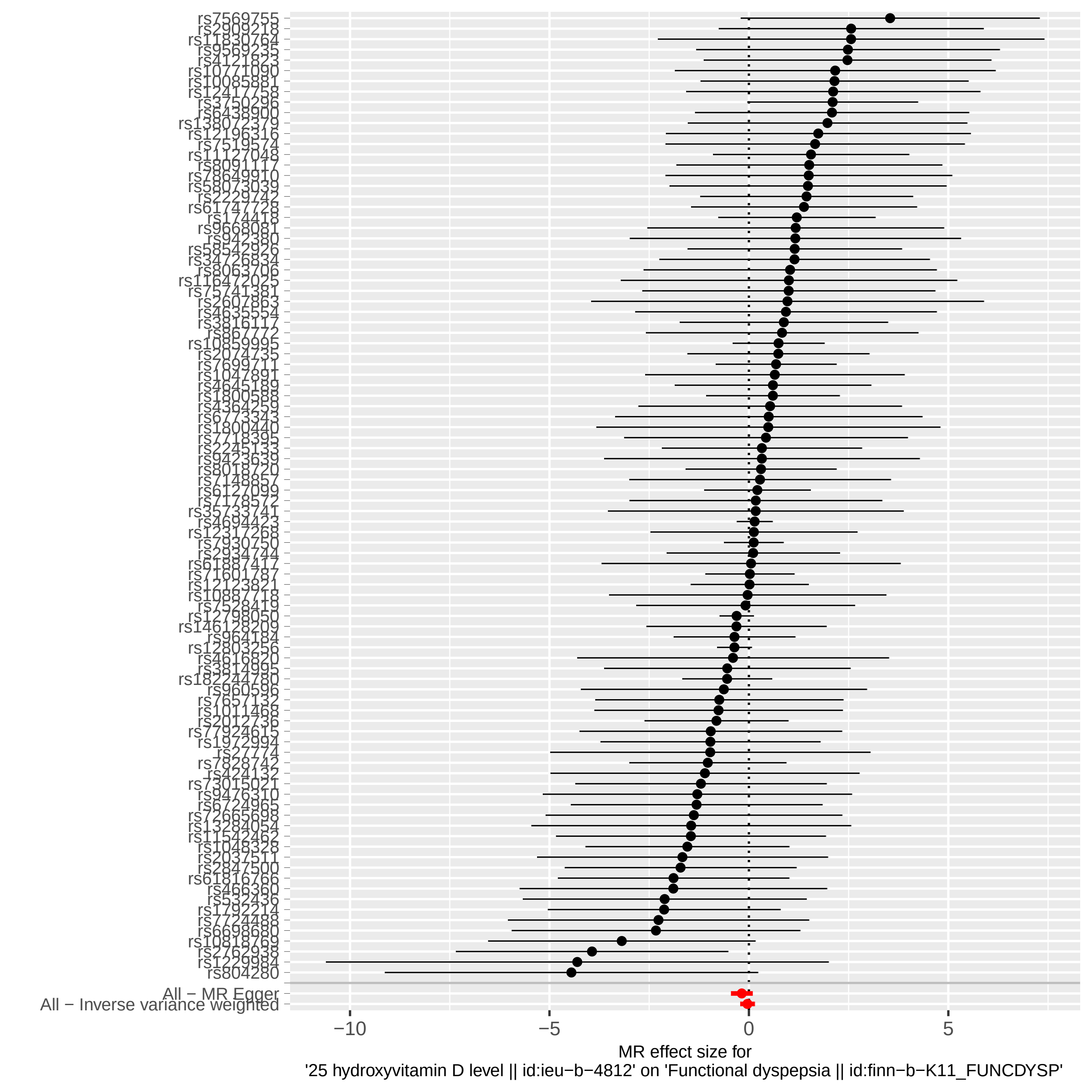
rs7569755
rs2909218
rs11830764
rs9569235
rs4121823
rs10771090
rs10085881
rs12417758
rs3750296
rs6438900
rs138072379
rs12196316
rs7519574
rs11127048
rs8091117
rs78649910
rs58073039
rs2229742
rs61747728
rs174418
rs9668081
rs942380
rs58542926
rs34726834
rs8063706
rs116472025
rs75741381
rs2607863
rs4635554
rs3816117
rs867772
rs10859995
rs2074735
rs7699711
rs1047891
rs4645189
rs1800588
rs4364259
rs6773343
rs1800440
rs7718395
rs2245133
rs9423639
rs8018720
rs7148857
rs6127099
rs7178572
rs35733741
rs4694423
rs12317268
rs7930750
rs2934744
rs61887417
rs71601787
rs12123821
rs10887718
rs7528419
rs12798050
rs146128209
rs964184
rs12803256
rs4616820
rs3814995
rs182244780
rs960596
rs7657132
rs1011468
rs2012736
rs77924615
rs1972994
rs27774
rs7828742
rs424132
rs73015021
rs9476310
rs6724965
rs72665698
rs13284054
rs11542462
rs1048328
rs2037511
rs2847500
rs61816766
rs466360
rs532436
rs1792214
rs7724488
rs6698680
rs10818769
rs2762938
rs1229984
rs804280
All − MR Egger
All − Inverse variance weighted
−10
−5
0
5
MR effect size for
'25 hydroxyvitamin D level || id:ieu−b−4812' on 'Functional dyspepsia || id:finn−b−K11_FUNCDYSP'
